# Decreased Histone Acetylation Levels at Th1 and Regulatory Loci after Induction of Food Allergy

**DOI:** 10.3390/nu12103193

**Published:** 2020-10-19

**Authors:** Bilal Alashkar Alhamwe, Laura A. P. M. Meulenbroek, Désirée H. Veening-Griffioen, Tjalling M. D. Wehkamp, Fahd Alhamdan, Sarah Miethe, Hani Harb, Astrid Hogenkamp, Léon M. J. Knippels, Elke Pogge von Strandmann, Harald Renz, Johan Garssen, Betty C. A. M. van Esch, Holger Garn, Daniel P. Potaczek, Machteld M. Tiemessen

**Affiliations:** 1 Institute of Laboratory Medicine, the German Center for Lung Research (DZL) and the Universities of Giessen and Marburg Lung Center (UGMLC), Philipps University Marburg, 35039 Marburg, Germany; bilal.alashkaralhamwe@staff.uni-marburg.de (B.A.A.); alhamdan@students.uni-marburg.de (F.A.); sarah.miethe@staff.uni-marburg.de (S.M.); hani.harb@childrens.harvard.edu (H.H.); renzh@med.uni-marburg.de (H.R.); garn@staff.uni-marburg.de (H.G.); danppot@gmail.com (D.P.P.); 2 Institute of Tumor Immunology, Clinic for Hematology, Oncology and Immunology, Center for Tumor Biology and Immunology, Philipps University Marburg, 35039 Marburg, Germany; poggevon@staff.uni-marburg.de; 3 College of Pharmacy, International University for Science and Technology (IUST), Daraa 15, Syria; 4 Danone Nutricia Research, 3584 CT Utrecht, The Netherlands; laura.meulenbroek@danone.com (L.A.P.M.M.); d.h.veening-griffioen@uu.nl (D.H.V.-G.); tjalling.wehkamp@danone.com (T.M.D.W.); leon.knippels@danone.com (L.M.J.K.); j.garssen@uu.nl (J.G.); e.c.a.m.vanesch@uu.nl (B.C.A.M.v.E.); 5 Division of Pharmacology, Utrecht Institute for Pharmaceutical Sciences, Faculty of Science, Utrecht University, 3584 CT Utrecht, The Netherlands; a.hogenkamp@uu.nl; 6 Translational Inflammation Research Division & Core Facility for Single Cell Multiomics, the German Center for Lung Research (DZL), Universities of Giessen and Marburg Lung Center, Philipps University Marburg, 35039 Marburg, Germany; 7 Division of Immunology, Boston Children’s Hospital, Harvard Medical School, Boston, MA 02115, USA; 8 John Paul II Hospital, 31-202 Krakow, Poland

**Keywords:** cow’s milk allergy, epigenetics, food allergy, histone acetylation, whey

## Abstract

Immunoglobulin E (IgE)-mediated allergy against cow’s milk protein fractions such as whey is one of the most common food-related allergic disorders of early childhood. Histone acetylation is an important epigenetic mechanism, shown to be involved in the pathogenesis of allergies. However, its role in food allergy remains unknown. IgE-mediated cow’s milk allergy was successfully induced in a mouse model, as demonstrated by acute allergic symptoms, whey-specific IgE in serum, and the activation of mast cells upon a challenge with whey protein. The elicited allergic response coincided with reduced percentages of regulatory T (Treg) and T helper 17 (Th17) cells, matching decreased levels of H3 and/or H4 histone acetylation at pivotal Treg and Th17 loci, an epigenetic status favoring lower gene expression. In addition, histone acetylation levels at the crucial T helper 1 (Th1) loci were decreased, most probably preceding the expected reduction in Th1 cells after inducing an allergic response. No changes were observed for T helper 2 cells. However, increased histone acetylation levels, promoting gene expression, were observed at the signal transducer and activator of transcription 6 (*Stat6*) gene, a proallergic B cell locus, which was in line with the presence of whey-specific IgE. In conclusion, the observed histone acetylation changes are pathobiologically in line with the successful induction of cow’s milk allergy, to which they might have also contributed mechanistically.

## 1. Introduction

Cow’s milk allergy (CMA), defined as an immune-mediated response to proteins in cow’s milk occurring consistently after ingestion, is one of the most common food allergies in early childhood, with the prevalence estimated to be around 0.5–3% in developed countries [1,2]. Although the data tend to vary between studies, about 60% of the patients suffering from CMA seem to be characterized by the type I hypersensitivity-based, immunoglobulin E (IgE)-mediated form of the disease [2,3,4].

Allergic disorders are considered prototypic examples of conditions determined by an interaction of the genetic background and environmental influences. The sharp increase in allergic diseases in a rather short time period is pointing towards the importance of environmental influences that are typically mediated by epigenetic mechanisms [5,6,7,8,9]. Not surprisingly, also in the case of CMA, while the genetic background is being further elucidated [10,11,12,13,14,15], more and more attention is now being paid to the role of epigenetics.

Epigenetics describes the activation and silencing of specific genes which involves microRNAs, DNA methylation, and histone acetylation. Although the contribution of microRNAs to the pathophysiology of CMA has been addressed [16], most of the epigenetic studies on CMA investigated DNA methylation [17,18,19,20]. Two of these studies used a targeted approach and studied the DNA methylation of specific genes in peripheral blood mononuclear cells (PBMCs). Berni Canani et al. showed a lower methylation of *Il4* and *Il5*; genes encoding interleukin-4 (Il-4) and -5 (Il-5), respectively, and a higher methylation of the gene encoding interleukin-10 (Il-10), *Il10*; and the gene encoding interferon γ, *Ifng*, in PBMCs of CMA individuals compared to controls [18]. Moreover, Paparo et al. investigated the demethylation of the regulatory T cell (Treg)-specific demethylated region in the gene encoding Foxp3 (*Foxp3)* in PBMCs and found a lower demethylation rate in CMA patients [17]. The lower methylation of *Il4* was confirmed by a large, untargeted study, in which the methylation status of more than 400,000 sites in whole blood DNA was investigated [19]. Interestingly, in the latter study, more hypomethylated positions were found in CMA patients compared to controls, whereas another untargeted study showed more hypermethylation [19,20]. More research is thus necessary to elucidate the role of DNA methylation in CMA.

To the best of our knowledge, the contribution of histone modifications to the development of CMA has not been investigated so far. Histone modifications can have an effect on gene expression. By making chromatin less dense and thus more accessible to the transcriptional machinery, higher levels of histone acetylation usually increase the chances of the genes for effective expression. Higher histone acetylation can thus be associated with enhanced transcriptional activity and increased gene expression; while lower histone acetylation can result in an opposite effect [5,6]. This may however not always be the case for histone H4 as partially opposing effects of H4 acetylation have also been reported [21,22,23]. A few studies have investigated the direct role of histone modifications in allergic disorders other than CMA. These studies clearly demonstrate the importance of this type of epigenetic modification in etiology and pathophysiology of allergies [24,25,26,27,28,29,30]. Whereas most of these studies investigated histone modification of genes involved in asthma, Harb et al. explored the acetylation status of several genes in the placenta and correlated these with the prevalence of sensitization in children [28]. Interestingly, a higher histone acetylation of *Ifng* was associated with a lower risk of sensitization to food, suggesting a role for histone modifications in food allergy development.

Therefore, in this study, we investigated how an allergic response in a murine CMA model affects the H3 and H4 histone acetylation levels at the promoter regions of the pivotal T cell and allergy-related B cell loci to clarify a possible role for this epigenetic mechanism in CMA.

## 2. Materials and Methods

### 2.1. Animals

Specific pathogen-free, five-week-old female C3H/HeOuJ mice with a minimum bodyweight of 16 g were obtained from Charles River Laboratories (Sulzfeld, Germany). Female mice were used as they are more sensitive for this model. Using mixed genders would increase the variation and thus the sample size. Upon arrival, mice were randomly allocated to the negative control (*n* = 7) or whey sensitization group (*n* = 14). Animals were housed at the animal facility of Utrecht University in conventional Makrolon^®^ type III cages (*n* = 7/cage) with standard chip bedding (LTE E-001, Abedd Vertriebs, Vienna, Austria). A facial tissue (Kimberley-Clark, Ede, The Netherlands) and a polycarbonate retreat, tunnel, and igloo (Datesand, Bredbury, UK) were added as cage enrichment. Mice were kept to a 12/12 h light/dark cycle (7 a.m. to 7 p.m./7 p.m. to 7 a.m.) and had unlimited access to water and food (cow’s milk free AIN-93G diet (cow’s milk proteins were replaced by soy proteins), ssniff Spezialdiäten, Soest, Germany [31]). Food pellets were refreshed weekly. Bodyweight of the mice was measured once a week to monitor health. No differences in weight between the groups were observed (data not shown). This study was conducted in accordance with institutional guidelines for the care and use of laboratory animals established by the Ethics Committee for Animal Experimentation of the University of Utrecht, and all animal procedures were approved under license of the national competent authority, securing full compliance to the European Directive 2010/63/EU for the use of animals for scientific purposes.

### 2.2. Experimental Design—Sensitization and Challenges

**Figure 1:**
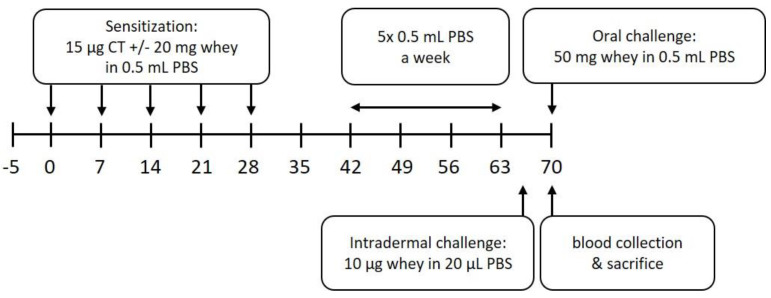
Schematic overview of the animal experiment. CT denotes cholera toxin, PBS denotes phosphate-buffered saline.

Mice were sensitized and treated as described previously [32]. In short, after an acclimatization period of 5 days, mice in the sensitization group were sensitized intragastrically (i.g.) with 20 mg whey protein (DMV International, Veghel, The Netherlands) and 15 µg cholera toxin (List Biological Laboratories, Campbell, CA, USA), which was used as an adjuvant, in 0.5 mL phosphate-buffered saline (PBS), once a week, for 5 consecutive weeks. The negative control group was treated at the same time with the same dose of cholera toxin in PBS. On day 35, blood was collected by a cheek puncture to determine the sensitization state. From day 42 until day 63, all animals were treated i.g. 5 consecutive days a week, for 3 weeks, with 0.5 mL PBS. To assess the allergic response, an intradermal (i.d.) challenge with whey protein (10 µg/20 µL PBS) in the ear pinnae of both ears was performed under isoflurane/air anesthesia on day 64, followed by an oral challenge (50 mg whey/0.5 mL PBS, i.g.) on day 70. Blood samples were collected 30 min after the oral challenge by terminal retro-orbital bleeding under isoflurane/air anesthesia (Abbott, Breda, The Netherlands) to determine serum levels of whey-specific IgE and mouse Mast Cell Protease-1 (mMCP-1). Immediately after the blood collection, mice were sacrificed by cervical dislocation and spleens were harvested to determine T cell subpopulations and the histone acetylation status of several loci in T and B cells. A schematic overview of the experimental procedures is depicted in Figure 1.

### 2.3. Assessment of the Acute Allergic Response

Acute allergic symptoms, such as the acute allergic skin response, anaphylactic shock symptoms, and body temperature, were assessed after the i.d. challenge in a blinded manner. For the acute allergic skin response, the basal ear thickness and the ear thickness 1 h after the challenge were measured under isoflurane/air anesthesia in duplicate for each ear using a digital micrometer (Mitutoyo, Veenendaal, The Netherlands). Mean values were calculated, and basal mean values were subtracted from the mean values 1 h after the challenge to determine the Δ ear swelling (µm). Anaphylactic shock symptoms were scored at 0, 15, 30, 45, and 60 min after the i.d. challenge using a previously described scoring table (Table 1) [33,34]. Body temperature was measured at 0, 30, and 45 min after the i.d. challenge using a rectal rodent probe (Braintree Scientific, Massachusetts, USA). If temperature dropped below 34 °C in combination with an anaphylactic shock score of II or III, mice were placed on heating pads to support recovery. In case the temperature dropped below 30 °C and/or an anaphylactic shock score of IV was obtained, the humane end point was reached, and mice were sacrificed by cervical dislocation.

### 2.4. Serum Levels of Whey-Specific IgE and mMCP-1

Blood was collected in MiniCollect^®^ tubes with clot activator and serum separator (Greiner Bio-One, Alphen aan de Rijn, The Netherlands) and stored on ice until further processing. The tubes were centrifuged for 10 min at 3000× *g*, after which the serum was collected, aliquoted, and stored at −20 °C. Whey-specific IgE levels and serum mMCP-1 concentrations were determined by means of ELISA. For the whey-specific IgE ELISA, 96 microwell plates (Nunc™, VWR International, Amsterdam, The Netherlands) were coated overnight at 4 °C with 100 µL/well whey protein (20 µg/mL) in carbonate/bicarbonate buffer (0.05 M, pH = 9.6). The next day, plates were washed and blocked at room temperature (RT) for 1 h with ELISA buffer (15 g/L human serum albumin (Sigma-Aldrich, Zwijndrecht, The Netherlands) in 0.05% Tween20/PBS, 200 µL/well). Samples and standards (pooled sera positive for whey-specific immunoglobulins) were diluted in ELISA buffer, added to the plates (100 µL/well), and incubated for 2 h at RT. Subsequently, plates were washed, incubated for 1.5 h at RT with 100 µL/well biotin-conjugated Rat Anti-Mouse IgE antibodies (1 µg/mL, BD Biosciences, San Jose, CA, USA), and washed again. Finally, the plates were incubated with streptavidin-horseradish peroxidase (10,000× dilution, 100 µL/well, Sanquin Reagents, Amsterdam, The Netherlands) for 1 h at RT in the dark, washed, and developed with TMB substrate (100 µL/well). The reaction was stopped with 10% H_2_SO_4_ solution (100 µL/well) after 2–15 min depending on the standard curve staining. Optical density (OD) was measured at 450 nm on a plate reader (Powerwave HT, Biotek, Winoonski, VT, USA) and converted to arbitrary units (AU). Only dilutions of which the OD values fell into the linear part of the standard curve were used for these calculations. If this was the case for multiple dilutions of the same serum sample, the mean level was calculated and depicted. Values below detection limit were set on half of the detection limit (6.2 AU). The mMCP-1 levels were measured using the mouse MCPT-1 (mMCP-1) ELISA Ready-SET-Go!^®^ kit (eBioscience, Breda, The Netherlands) according to the manufacturer’s instructions. Data was analyzed in a similar way as the IgE ELISA. Only dilutions of which the OD values fell into the linear part of the standard curve were used for the calculations and values below detection limit were set on half of the detection limit (2.34 ng/mL).

### 2.5. Flow Cytometry Analysis

To determine the T cell subsets, spleens were collected 30 min after the oral challenge and stored in culture medium (RPMI 1640, 5% heat-inactivated fetal bovine serum (FBS), Penicillin (50 U/mL)-Streptomycin (50 µg/mL), all Gibco™, Thermo Fischer Scientific, Lansmeer, The Netherlands) on ice until further processing. Single cell suspensions were obtained by dissociating the spleens in 5 mL ice-cold lysis buffer (8.3 g/L NH_4_Cl, 1 g/L KHCO_3_ and 37.2 mg/L Na_2_EDTA, adjusted to pH 7.3) in gentleMACS C Tubes (Miltenyi Biotec, Leiden, The Netherlands) with a gentleMACS Octo Dissociator (program m_spleen_01, Miltenyi Biotec). The dissociated cell suspensions were applied to cell strainers and single cells were collected in culture medium. Cells were washed (5 min, 1500 rpm, 4 °C), counted, and plated at 1 × 10^6^ cells/well in V-bottom 96 well-plates. Subsequently, cells were washed in PBS and stained with Fixable Viability Dye eFluor 506 (eBioscience) for 30 min at 4 °C. After washing with FACS buffer (2% FBS and 2 mM EDTA in PBS), cells were blocked with purified Rat Anti-Mouse CD16/32 (1:100, 25 µL/well, Becton Dickinson) for 20 min at 4 °C and washed again. Cells were stained with 25 µL/well of either a T helper 1 (Th1)/T helper 2 (Th2) surface staining mix (FITC-conjugated Rat Anti-Mouse T1ST2 (1:100, clone DJ8, MD Bioproducts, Zürich, Switzerland); PerCP-Cy5.5-conjugated Rat Anti-Mouse CD4 (1:100, clone RM4-5, eBioscience); APC-Cy7-conjugated Rat Anti-Mouse CD8a (1:100, clone 53-6.7, BD Biosciences); and PE-Cy7-conjugated Armenian hamster Anti-Mouse CD69 (1:100, clone H1.2F3, eBioscience)) or a Treg/T helper 17 (Th17)/Th2 surface staining mix (PerCP-Cy5.5-conjugated Rat Anti-Mouse CD4; APC-Cy7-conjugated Rat Anti-Mouse CD8a; and PE-Cy7-conjugated Rat Anti-Mouse CD25 (1:100, clone PC61, BD Bioscience)) for 30 min at 4 °C. Cells were washed again and fixed overnight in Fixation buffer (Fixation/Permeabilization Concentrate, eBioscience). The next day, cells were permeabilized with Permeabilization buffer (Fixation/Permeabilization Concentrate, eBioscience) for 10 min to 18 h at 4 °C. Permeabilized cells were blocked again with purified Rat Anti-Mouse CD16/32 and washed (5 min, 2500× *g*, 4 °C), after which the cells were stained with 25 µL/well of either a Th1/Th2 intracellular staining mix (PE-conjugated Rat Anti-Mouse Gata3 (1:25, clone TWAJ, eBioscience) and eFluor^®^ 660-conjugated Mouse Anti-Mouse T-Box 21 (1:100, clone, eBio4B10, eBioscience)) or a Treg/Th17/Th2 intracellular staining mix (FITC-conjugated Rat Anti-Mouse Foxp3 (1:100, clone FJK-16s, eBioscience), PE-conjugated Rat Anti-Mouse RORγ(t) (1:100, clone AFKJS-9, eBioscience) and eFluor^®^ 660-conjugated Rat Anti-Mouse Gata3 (1:100, clone TWAJ, eBioscience)) for 30–60 min at 4 °C. After washing, the cells were fixed (100 µL/well, BD cellFIX) and analyzed using BD FACS Canto II and Flow Jo V.10.1 (all BD Bioscience).

### 2.6. Chromatin Immunoprecipitation to Determine Histone Acetylation Status in Splenocyte-Derived CD4^+^ T and B Cells

After the challenge, at day 70, CD4^+^ T cells and B cells deriving from whey- and PBS-treated mice were isolated from splenocytes using MACS, according to the manufacturer’s instructions (Miltenyi Biotec). Isolated CD4^+^ T cells and B cells were frozen in heat-inactivated FBS with 15% dimethyl sulphoxide (DMSO, Carl Roth, Karlsruhe, Germany) and stored in liquid nitrogen until further processing. Detailed methodology of chromatin immunoprecipitation (ChIP) followed by quantitative polymerase chain reaction (qPCR) along with its thoughtful validations are described in more detail elsewhere [35,36]. In brief, cross-linking of the cells was performed by incubating them for 8 min with paraformaldehyde (PFA; Carl Roth) to a final concentration of 1% at room temperature. The reaction was quenched by adding glycine (Carl Roth) to a final concentration of 125 mM. Following centrifugation at 8000 rpm for 5 min at 4 °C and washing with cold PBS, samples were subjected to 20 min of incubation with lysis buffer I (Table S1) at 4 °C. Lysis buffer II (Table S1) with 1% sodium dodecyl sulfate (SDS; Carl Roth) was added for 5 min at 4 °C. Thereafter, shearing of the DNA–protein complexes with the Bioruptor (Diagenode, Liège, Belgium) was performed using 30 cycles of 30-s on and 30-s off. The interfering debris was then removed by centrifugation at 15,000 rpm for 15 min at 4 °C. Sepharose beads (GE Healthcare Biosciences, Uppsala, Sweden) were first washed with lysis buffer II with 0.1% SDS. Following centrifugation at 3000 rpm for 2 min at room temperature, the beads were blocked with 1 mg/mL bovine serum albumin (BSA; Sigma-Aldrich) and 40 µg/mL salmon sperm DNA (Sigma-Aldrich) overnight at 4 °C. After washing such prepared beads with lysis buffer II with 0.1% SDS and centrifugation at 3000 rpm for 5 min at 4 °C, 30 µL of beads slurry per immunoprecipitation (IP) per number of samples were stored at 4 °C for the next day. To perform chromatin preclearing, 20 µL of beads slurry per antibody were added to the previously cross-linked chromatin samples, incubated with rotation for 2 h at 4 °C, and finally centrifuged at 8000 rpm for 5 min at 4 °C. To the remaining beads, 500 µL of lysis buffer II with 0.1% SDS and 1 µg of unspecific IgG (Abcam, Cambridge, UK) per sample were added and incubated with rotation for 1 h at 4 °C. After washing 3 times with lysis buffer II with 0.1% SDS, 20 µL of the IgG-coupled beads were added to the precleared chromatin, incubated with rotation for 2 h at 4 °C, and then centrifuged at 8000 rpm for 5 min at 4 °C. Ten percent of the resulting supernatant containing chromatin were stored as the input control. The rest was divided into equal parts, to which 4 µg of either H3 or H4 (Millipore, Darmstadt, Germany) or 0.5 µg of IgG (Abcam) were added. The samples were then incubated at 4 °C overnight. Thirty microliters of the blocked beads slurry kept aside before were added to each IP and incubated for 2 h at 4 °C. After centrifugation at 8000 rpm for 5 min at 4 °C, the beads were washed twice with wash buffer I, twice with lysis buffer II, three times with wash buffer III (Table S1), and finally, twice with TE buffer with pH 8.0 (Table S1). Elution of the chromatin was performed by adding 500 µL of elution buffer (Table S1) to the Sepharose beads, vortexing, and incubating with rotation for 30 min. After centrifugation at 8000 rpm for 2 min at 4 °C, the supernatants containing each IP, as well as the input controls, were mixed with 20 µL of 5 M NaCl, 10 µL of 0.5 M EDTA (Sigma-Aldrich), 20 µL of 1 M Tris-HCl (pH 7.2), 1 µL of Protease K (20 mg/mL; Sigma-Aldrich), and 1 µL of RNase A (10 mg/mL; Sigma-Aldrich) per sample. All samples were incubated at 55 °C for 3 h and, afterwards, at 65 °C overnight. Thereafter, DNA was purified using the QIAquick PCR purification kit (Qiagen, Hilden, Germany). The purified DNA was subjected to qPCR performed with specific mouse gene promoter primers (Table S2) and Rotor-Gene SYBR Green PCR Kit (Qiagen), conducted on Rotor-Gene Q (Qiagen). Percent enrichment to the input was calculated using the following formula: ${\%\;\mathrm{enrichment}\;=\;100\;\times \;2}^{\left[\left(\mathrm{CT}\;\mathrm{input}\text{-}3.3\right)\text{-}\mathrm{CT}\;\mathrm{sample}\right]}$. Then, the % enrichment of the isotype (IgG) control was subtracted from % enrichments obtained for specific antibodies. For final normalization, such value obtained for each specific gene was divided by that of the positive control gene *Rpl32*.

### 2.7. Statistical Analysis

Groups reported here were the control groups of a larger oral immunotherapy study in which the sample size was determined based on the acute allergic skin response, the primary outcome of that study. The following numbers were used for the sample size calculation: for the control group, α = 0.025, power = 90%, σ = 17%, and effect size = 31%; for the whey group, α = 0.00125, power = 90%, σ = 17%, and effect size = 25%. Single animals were considered as experimental unit. To correct for possible dropouts due to reaching humane endpoint after the i.d. challenge, 10% extra animals were included in the whey group. However, none of the mice reached the humane endpoint in this group.

Statistical analyses were performed using GraphPad Prism software (version 8.3.1; GraphPad Software, San Diego, CA, USA). Data are expressed as scatter dot plot showing individual data points with mean/median. Between-groups comparisons were conducted with unpaired t test, Welch’s t test, or Mann–Whitney U-test. The unpaired T test was selected if the data were normally distributed (if possible, tested with D’Agostino and Pearson normality test) and had an equal σ. If data was not normally distributed based on the normality test or previous experiences with larger sample sizes, the Mann–Whitney U-test was performed. The Welch’s t test was performed when the σ of the groups was not equal. Results were considered statistically significant when *p* < 0.05. *p* values between 0.05 and 0.2 were considered trends.

## 3. Results

### 3.1. Whey Protein Induces Food Allergy in a Murine Cow’s Milk Allergy Model

To induce CMA, mice were sensitized i.g. with a combination of whey protein and cholera toxin as adjuvant, as described previously [32]. Mice in the control group were treated with cholera toxin in PBS only. The allergic response measured as an acute skin response and anaphylactic symptoms were determined after an i.d. challenge (D64) with whey protein. Subsequently, an oral challenge (D70) with whey protein was used to investigate serum Immunoglobulins and mMCP-1 levels (Figure 1).

**Figure 2:**
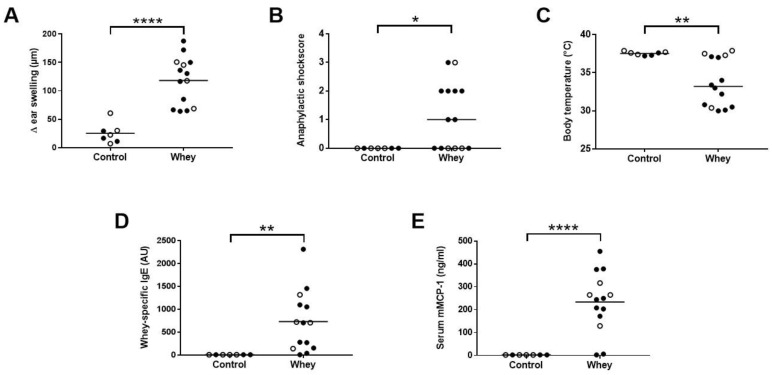
Acute allergic symptoms upon whey protein challenge in a cow’s milk allergy model. (**A**) The acute allergic skin response measured as Δ ear swelling 1 h after intradermal (i.d.) challenge. (**B**) Anaphylactic shock scores and (**C**) body temperature determined 30 min after i.d. challenge. (**D**) Whey-specific IgE and (**E**) mouse mast cell protease-1 (mMCP-1) levels in serum collected 30 min after the oral challenge. Data are presented as scatter dot plots showing individual data points with mean (**A**,**D**,**E**) or median (**B**,**C**). Open circles indicate the mice that were included in the histone modification analysis. Comparisons were performed with an unpaired t test (**A**), Welch’s t test (D,E) or Mann–Whitney U test (**B**,**C**). * *p* < 0.05, ** *p* < 0.01, **** *p* < 0.0001.

As expected, whey-sensitized mice showed a significant increase in the acute allergic skin response after i.d. whey challenge compared to control mice (Figure 2A). In addition, an increase in anaphylactic shock symptoms and, as a result, a significant drop in body temperature were observed in whey-sensitized mice (Figure 2B,C). Serum analysis after the oral challenge showed a significant increase in whey-specific IgE (Figure 2D) and mMCP-1 (Figure 2E) compared to controls, indicating the activation and degranulation of mast cells.

### 3.2. A Confirmed Allergic Response to Whey Protein Was Not Associated with an Increase in Spleen Th2 Cells and Histone Acetylation Changes at Th2 Loci

**Figure 3:**
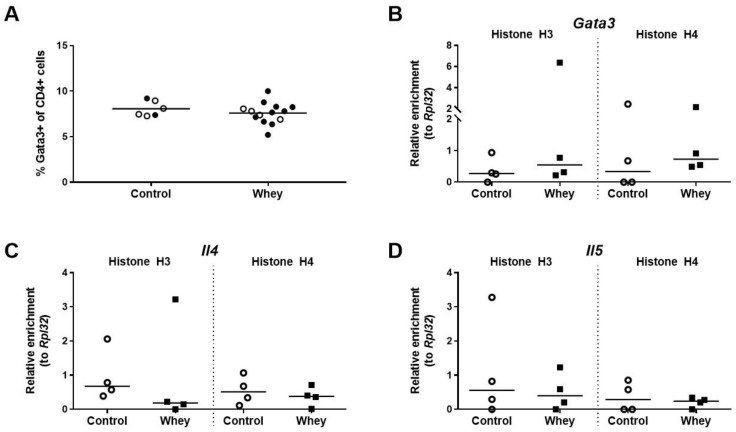
A confirmed allergic response to whey protein affected neither the percentages of Th2 cells in the spleen nor the H3 and H4 histone acetylation levels of Th2-related genes in the total CD4^+^ T cell population in the spleen. (**A**) The percentage of CD4^+^ T cells positive for GATA binding protein 3 (Gata3) in the spleen as assessed by flow cytometry. Data are presented as a scatter dot plot showing individual data points and mean. Open circles indicate the mice that were included in the histone modification analysis. Data of one mouse in the control group was excluded due to a technical error. Comparisons were performed with an unpaired t test. The histone acetylation levels at the Gata3 gene (*Gata3*) (**B**), the interleukin 4 gene (*Il4*) (**C**), and the interleukin 5 gene (*Il5*) (**D**) in the total CD4^+^ T cell population in the spleen as determined by chromatin immunoprecipitation followed by qPCR. Data are presented as scatter dot plots showing individual data points and median. Comparisons were performed with a Mann–Whitney U test.

The percentage of splenic CD4^+^ T cells having a Th2 phenotype, as assessed by positivity for GATA binding protein 3 (Gata3), a Th2 lineage master regulator [37], was not different between whey-sensitized mice and control mice after the whey protein challenge (Figure 3A). The effect of the whey challenge on the levels of histone acetylation at the promoter regions of the gene encoding Gata3 (*Gata3*) was investigated in the total CD4^+^ T cell population. *Il4* and *Il5,* the genes encoding the two hallmark Th2 cytokines, were studied as well. No difference was observed between whey-sensitized and control mice for the levels of histone acetylation on any of the three genes tested (Figure 3B–D).

### 3.3. A Confirmed Allergic Response to Whey Protein Had No Effect on the Percentage of Spleen Th1 Cells but Decreased Histone Acetylation Levels at the Gene Encoding T-Box 21

**Figure 4:**
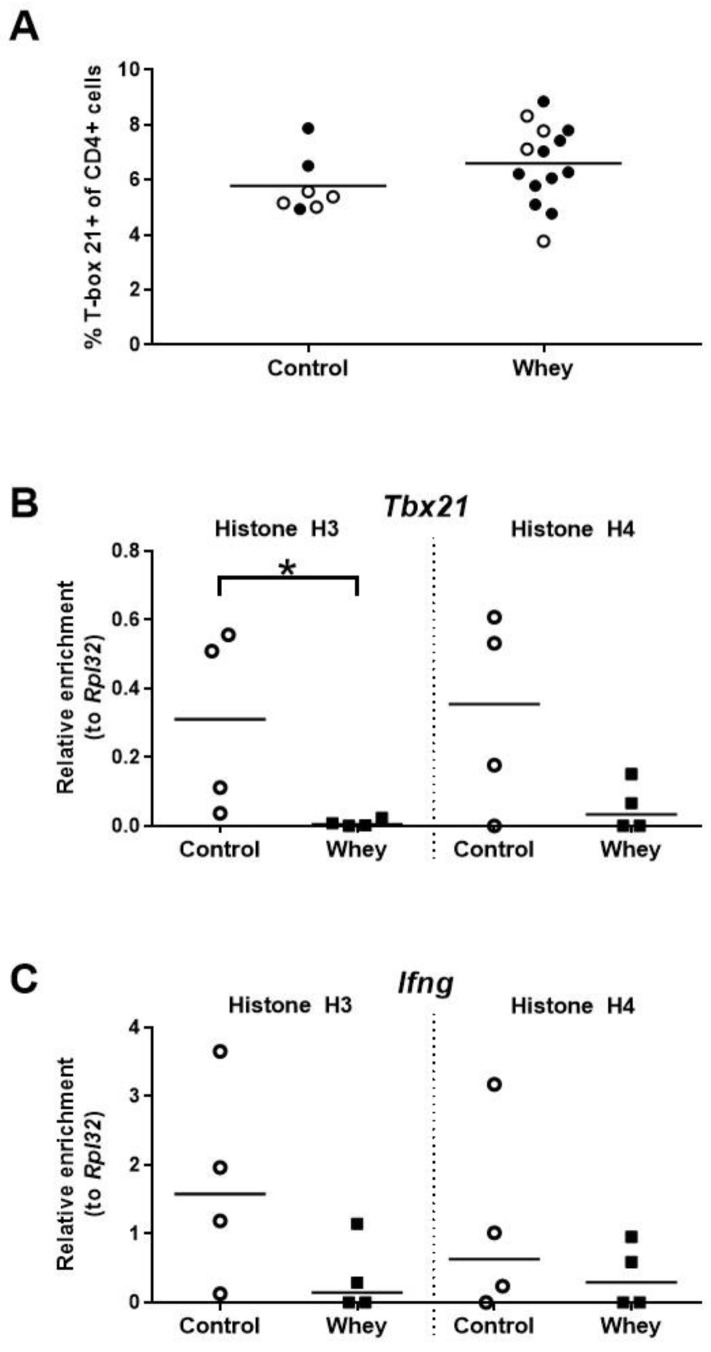
A confirmed allergic response to whey protein was associated with an unaltered percentage of Th1 cells in the spleen and decreased H3 histone acetylation levels at a gene encoding T-box 21 in spleen-derived CD4^+^ T cells. (**A**) The percentage of CD4^+^ T cells positive for T-box 21 (T-bet) as assessed by flow cytometry. Data are presented as a scatter dot plot showing individual data points and mean. Open circles indicate the mice which were included in the histone modification analysis. Comparisons were performed with an unpaired t test. Histone acetylation levels at the T-box 21 gene (*Tbx21*) (**B**) and the interferon γ gene (*Ifng*) (**C**) in spleen-derived CD4^+^ T cells as determined by chromatin immunoprecipitation followed by qPCR. Data are presented as scatter dot plots showing individual data points and median. Comparisons were performed with a Mann–Whitney U test. * *p* < 0.05.

The percentage of Th1 cells in the spleen—as assessed by positivity for T-box 21 (Tbx21; also called T-bet), a master transcription factor of the Th1 lineage [38]—was not different between whey-sensitized mice and control mice upon whey protein challenge (Figure 4A). Interestingly, the induction of an allergic response against whey protein was associated with a significant decrease in histone H3 acetylation at *Tbx21*, a gene encoding Tbx21 (Figure 4B). However, no significant difference was seen for histone H4 (Figure 4B). In addition, no significant differences between whey-sensitized and control mice were demonstrated for histone acetylation levels at the promoter of *Ifng*, a gene encoding the major Th1 cytokine (Figure 4C).

### 3.4. A Confirmed Allergic Response to Whey Protein Reduced the Numbers of Spleen Th17 Cells and Tended to Decrease Histone Acetylation Levels at RORγt

**Figure 5:**
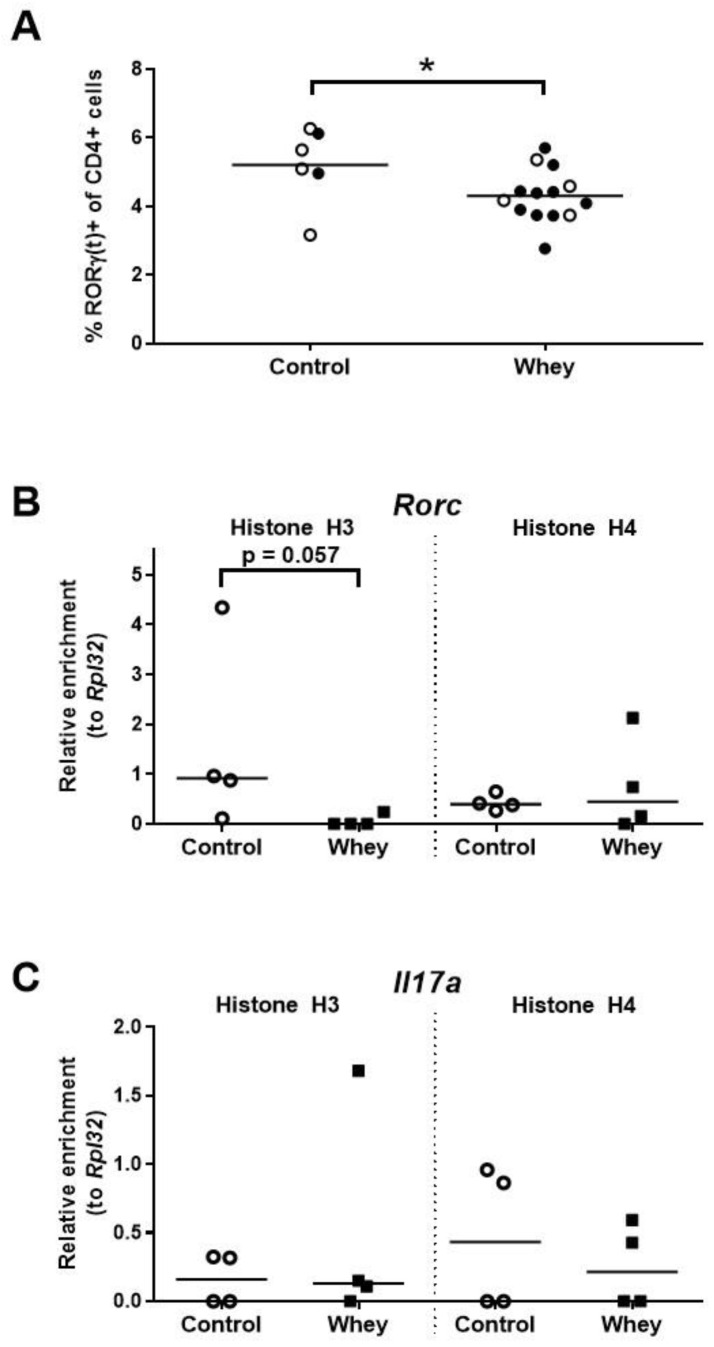
A confirmed allergic response to whey protein reduced the percentages of spleen Th17 cells and tended to decrease H3 histone acetylation levels at the promoter responsible for transcription of RORγt. (**A**) The percentage of CD4^+^ T cells positive for RORγ(t) as assessed by flow cytometry. Data are presented as a scatter dot plot showing individual data points and mean. Open circles indicate the mice that were included in the histone modification analysis. Data of one mouse in the control group were excluded due to a technical error. Comparisons were performed with an unpaired t test. Histone acetylation levels at the promoter responsible for the transcription of RORγt (i.e., isoform 2) from the RAR-related orphan receptor γ encoding gene (*Rorc*) (**B**) and the interleukin 17A gene (*Il17a*) (**C**) in spleen-derived CD4^+^ T cells as determined by chromatin immunoprecipitation followed by qPCR. Data are presented as scatter dot plots showing individual data points and median. Comparisons were performed with a Mann–Whitney U test. * *p* < 0.05; *p*-values ≥ 0.05 and <0.2 are shown as numbers.

In whey-sensitized mice, the percentage of spleen Th17 cells—as assessed by positivity for RORγt, a master regulator of the Th17 lineage [39,40]—was found to be lower than in controls (Figure 5A). Additionally, a tendency towards lower histone H3 acetylation levels were found in the whey-sensitized mice as compared to control mice at the promoter responsible for the transcription of RORγt (i.e., isoform 2) from the RAR-related orphan receptor γ encoding gene (*Rorc*; Figure 5B). Histone acetylation levels at the interleukin 17A (Il17a) encoding gene (*Il17a*) remained, however, unaffected (Figure 5C).

### 3.5. A Confirmed Allergic Response to Whey Protein Tended to Reduce the Percentage of Treg Cells in the Spleen and Decreased Histone Acetylation Levels at the Promoter of the Interleukin 10 Gene

**Figure 6:**
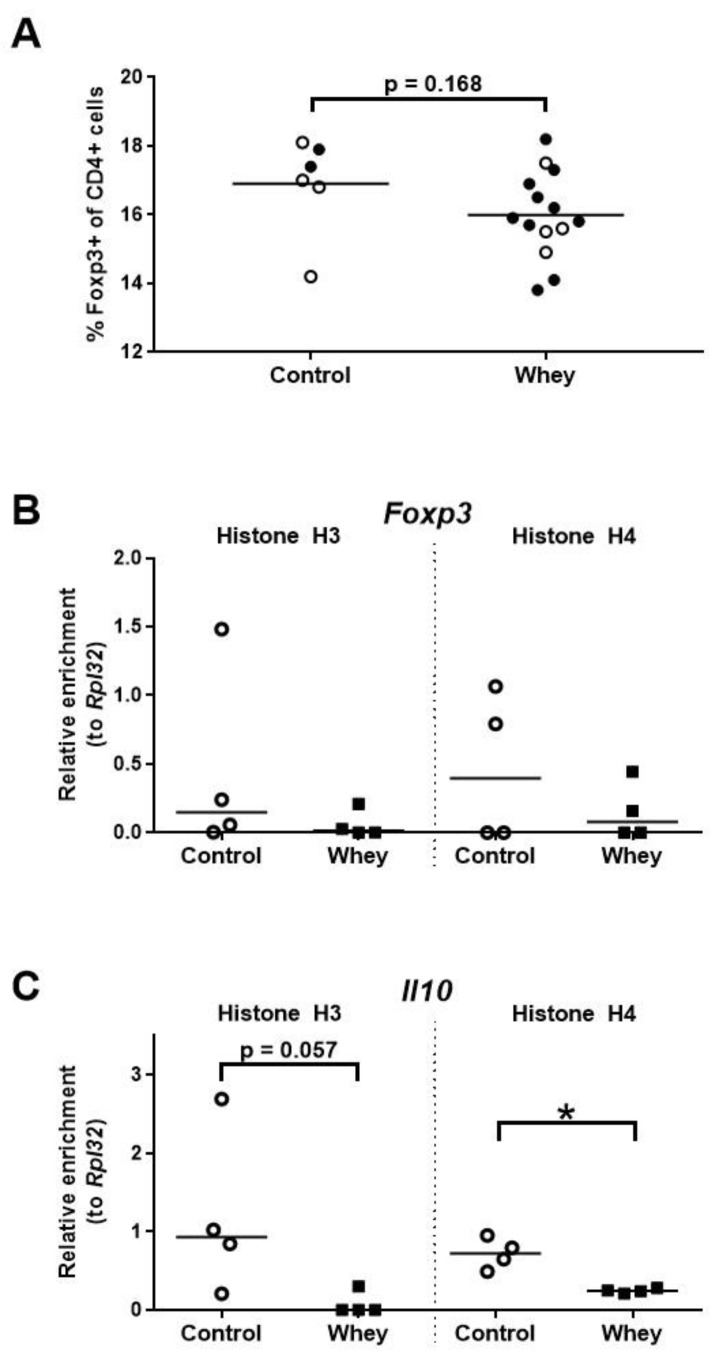
A confirmed allergic response to whey protein was associated with a tendency towards a lower percentage of spleen Treg cells and decreased H4 histone acetylation levels at the interleukin 10 gene. (**A**) The percentage of CD4^+^ T cells positive for forkhead box P3 (Foxp3) as assessed by flow cytometry. Data are presented as a scatter dot plot showing individual data points and mean. Open circles indicate the mice that were included in the histone modification analysis. Data of one mouse in the control group was excluded due to a technical error. Comparisons were performed with an unpaired t test. Histone acetylation levels at the Foxp3 gene (*Foxp3*) (**B**) and the interleukin 10 gene (*Il10*) (**C**) in spleen-derived CD4^+^ T cells as determined by chromatin immunoprecipitation followed by qPCR. Data are presented as scatter dot plots showing individual data points and median. Comparisons were performed with a Mann–Whitney U test. * *p* < 0.05; *p*-values ≥ 0.05 and <0.2 are shown as numbers.

Analysis of the percentages of the CD4^+^ splenocytes characterized by a Treg phenotype—as assessed by positivity for forkhead box P3 (Foxp3), a Treg lineage master transcription factor [41,42]—demonstrated a tendency towards a lower percentage of Tregs in whey-sensitized mice versus control mice (Figure 6A). However, no effect was seen on the histone acetylation pattern of *Foxp3* (Figure 6B). The levels of histone acetylation at the promoter of *Il10*, a gene encoding the hallmark Treg cytokine, tended to be lower in the whey-sensitized mice than in the controls (Figure 6C).

### 3.6. A Confirmed Allergic Response to Whey Protein Increased Histone Acetylation Levels at the Promoter of Stat6 but Had No Effect on Other Allergy-Related B Cell Loci

**Figure 7:**
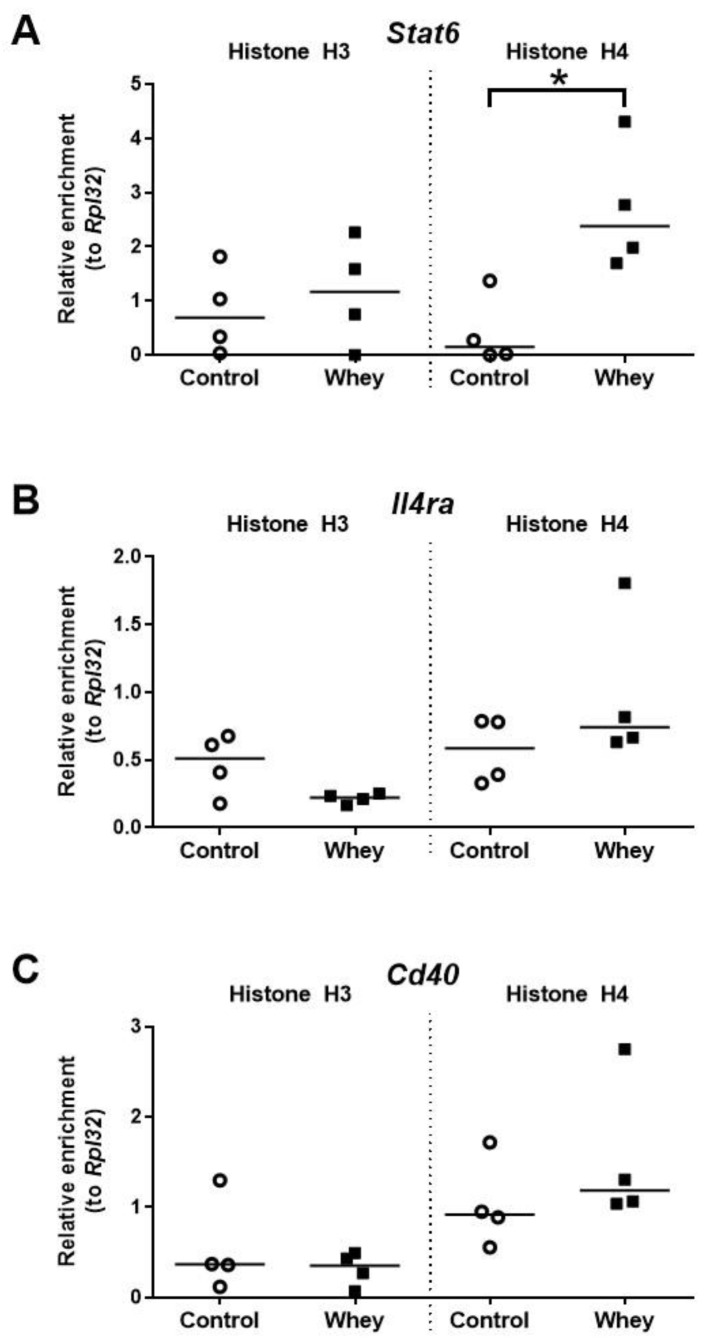
A confirmed allergic response to whey protein increased H4 histone acetylation levels at the promotor of *Stat6* but had no effect on other allergy-related B cell genes. Histone acetylation levels at the signal transducer and activator of transcription 6 gene (*Stat6*) (**A**), the interleukin 4 receptor alpha gene (*Il4ra*) (**B**), and the CD40 antigen gene (*Cd40*) (**C**) in spleen-derived B cells as determined by chromatin immunoprecipitation followed by qPCR. Data are presented as scatter dot plots showing individual data points with median. Comparisons were performed with Mann–Whitney U-test; * *p* < 0.05.

The histone H4 acetylation levels at the promoter of *Stat6*—a gene encoding signal transducer and activator of transcription 6—were significantly higher in spleen-derived B cells obtained from whey-sensitized mice compared to control mice upon challenge (Figure 7A). No effects were observed for the histone acetylation levels at the promoters of *Il4ra*, a gene encoding interleukin 4 receptor alpha (Il4ra); and *Cd40*, a gene encoding CD40 antigen (Cd40) (Figure 7B,C).

## 4. Discussion

Histone acetylation is an important epigenetic mechanism and can affect the pathogenesis of allergies. The present study demonstrates for the first time changes in histone acetylation patterns at important T and B cell genes after an allergic response to a food protein—in this case, whey protein—in C3H/HeOuJ mice.

The induction of an allergic response against whey protein was successful in our model, as evidenced by the presence of acute allergic symptoms and mast cell activation (mMCP-1) in sensitized and subsequently challenged mice. The observed allergic symptoms were most likely IgE-mediated, as reflected by high levels of whey-specific IgE in the whey-sensitized animals.

The allergic response in this study is not associated with an increase in the histone acetylation levels at pivotal Th2 genes such as *Il4*, *Il5*, and *Gata3*. One would expect an increase in activation of Th2-mediated genes, considering their crucial role in IgE-mediated hypersensitivity [43,44]. It is possible that dynamic changes in histone acetylation levels across the loci encoding Th2 genes are only temporarily demonstrated during the sensitization phase of the allergic response and that epigenetic mechanisms are less dominant in the challenge phase of the allergic response. Unfortunately, histone acetylation levels were only measured after induction of the allergic response and not directly after sensitization.

Whilst no changes were demonstrated for the Th2 genes, a decrease in histone acetylation at *Tbx21*, an important Th1 gene, was detected. The observed histone acetylation changes at this gene may precede a potential downregulation of Th1 gene expression and a subsequent reduction of Th1 cell numbers or activities. This decrease may thus contribute to a skewed type-1/type-2 balance towards proallergic type-2 responses. Indeed, the ratio of histone H3 acetylation at promoters of *Gata3* and *Tbx21* turned out to be significantly higher in whey-sensitized mice compared to control mice upon challenge (Figure S1).

Interestingly, changes in spleen-derived T cell populations and the epigenetic status of the hallmark genes associated with the allergic response were rather consistent for the Treg lineage. The percentage of Tregs in the spleen tended to be lower in whey-sensitized mice compared to control mice. At the same time, a reduction in histone acetylation at the promoter of *Il10,* a hallmark cytokine for Tregs, was observed. Considering that induction and maintenance of tolerance to food allergens requires the generation of allergen-specific Treg cells [45,46,47], it could be hypothesized that Treg-related histone acetylation changes as well as their functional consequences reflect the disruption in the process of oral tolerance, and hence, promote the allergic response.

The role of Th17 immunity in food allergy is less clearly defined than in the case of asthma, where it is known to contribute to the pathophysiology of non-type-2 phenotypes of the disease [48,49,50,51,52,53]. A protective role of Th17 cells against food allergy, with the defect in Th17 immunity as a prerequisite for the development of food allergy, has been postulated [49]. Moreover, a recent study indicated that the number of RORγt^+^ Treg cells was reduced in food allergic subjects [54]. In whey-sensitized mice, we observed a reduction in histone H3 acetylation levels at the promoter responsible for transcription of RORγt accompanied by a decrease in the percentage of RORγt^+^ T cells in the spleen. Thus, our findings seem to be in line with the postulated tolerogenic role of RORγt^+^ T cells in food allergy.

The epigenetic changes observed by us in B cells seem to be directly in line and may mechanistically support the development of IgE-mediated allergic response in the whey-sensitized mice. Stat6 is a transcription factor that plays a pivotal role in mediating class-switch recombination towards IgE, a process during which B cells gain the ability to synthesize IgE, and other molecular events enabling IgE production. Stat6 is activated in B cells after the stimulation with type-2 cytokine Il4. The second signal for IgE production is provided by an interaction of B cell surface Cd40 with its ligand Cd40l(g) [43,55]. In this study, we observed significantly higher histone H4 acetylation levels at *Stat6* in whey-sensitized mice, but we found no differences in histone acetylation levels at *Il4ra* and *Cd40* genes between sensitized and control mice. This fits with our hypothesis that histone acetylation changes in Th2 genes can only be temporarily demonstrated during the sensitization phase and cannot be detected after a challenge as the stimulation of the Il4r and Cd40 is directly downstream of the Th2 cell activation. In contrast, the activation of Stat6 seems to be either later or more long-lasting and can be detected upon challenge.

The spectrum of the epigenetic effects associated with the consumption of cow’s milk extends, however, beyond the contribution of histone acetylation to the induction of CMA [56]. It has been shown that early life exposure to raw farm milk, i.e., maternal and/or early childhood unprocessed farm milk consumption, protects against the development of asthma and other allergies, and that these effects seem to be mediated in part through epigenetic mechanisms [57]. The importance of DNA demethylation at the *Foxp3* gene with activation of Treg cells has been demonstrated in these studies [58,59], as well as the role of histone acetylation changes at important T cell genes in mouse experiments [36]. Interestingly, in addition to the classical epigenetic mechanisms, raw farm milk can protect against allergy development through its own, milk-derived, immune-regulatory, exosomal microRNAs [60,61,62].

Our study has several limitations. First, epigenetic mechanisms different from histone acetylation, such as DNA methylation, other types of histone modifications, or microRNAs, may contribute to the epigenetic regulation of T and B cell genes participating in food allergy induction. However, this does not affect the potential regulatory picture emerging from our results. Rather, the contribution of other epigenetic pathways would be expected to fill the gaps in this picture, such as mechanisms of Th2 upregulation or those downstream of *Tbx21* or upstream of *Il10* in T cells, as potentially exemplified by the above-described DNA demethylation at the *Foxp3* in case of the latter. Secondly, the numbers of animals included in the epigenetic part of our investigation are limited. Further studies, integrating different epigenetic mechanisms and involving larger groups of animals investigated at several time points, corresponding to their experimental complexity as well as respective human investigations, should be planned and performed.

In conclusion, the observed histone acetylation changes are pathobiologically in line with the successful induction of cow’s milk allergy, to which they might have also contributed mechanistically.

## Figures and Tables

**Table 1:** Anaphylactic shock symptom scoring table.

Score	Symptoms
0	No symptoms
1	Scratching nose and mouth
2	Swelling around the eyes and mouth; pillar erect; reduced activity; higher breathing rate
3	Shortness of breath; blue rash around the mouth and tail; higher breathing rate
4	No activity after stimulation; shivering and muscle contractions
5	Death by shock

## Supplementary Materials

The following are available online at https://www.mdpi.com/2072-6643/12/10/3193/s1, Table S1: Buffers used for ChIP, Table S2: Primers used for qPCR, Figure S1: A confirmed allergic response to whey protein increased the ratio of histone acetylation levels at the gene encoding Gata3 (*Gata3*) and the gene encoding T-box 21 (*Tbx21*) in spleen-derived CD4^+^ T cells.
