# Supplementary material for: Decreased Histone Acetylation Levels at Th1 and Regulatory Loci after Induction of Food Allergy
Source: Nutrients. 2020 Oct 19;12(10):3193. doi: 10.3390/nu12103193 (PMC7603208; doi:10.3390/nu12103193)
Supplement: Supplementary file 1 [file nutrients-12-03193-s001.pdf]

Table S1. Buffers used for ChIP.

**Lysis buffer I**	For 50 mL
5 mM PIPES pH 8	0.5 mL 0.5 M PIPES pH 8
85 mM KCl	1.4 mL 3 M KCl
0.5% NP40 (Igepal-CA630)	0.25 mL Igepal (100%)
Protease inhibitor cocktail tablets	One tablet

**Lysis buffer II**	For 50 mL
10 mM Tris-HCl pH 7.5	0.5 mL 1 M Tris-HCl pH 7.5
150 mM NaCl	1.5 mL 5 M NaCl
1% NP40 (Igepal-CA630)	0.5 mL Igepal (100%)
1% DOC (Natriumdeoxycholat)	0.5 g
0.1% SDS	0.25 mL 20% SDS
1 mM EDTA	0.1 mL 0.5 M EDTA pH 8
Protease inhibitor cocktail tablets	One tablet

**Wash buffer I**	For 50 mL
20 mM Tris-HCl pH 8	1 mL Tris-HCl pH 8
150 mM NaCl	1.5 mL 5 M NaCl
2 mM EDTA	0.2 mL 0.5 M EDTA pH 8
0.1% SDS	0.25 mL 20% SDS
1% Triton X100	0.5 mL Triton X100

**Wash buffer II**	For 50 mL
20 mM Tris-HCl pH 8	1 mL Tris-HCl pH 8
500 mM NaCl	5 mL 5 M NaCl
2 mM EDTA	0.2 mL 0.5 M EDTA pH 8
0.1% SDS	0.25 mL 20% SDS
1% Triton X100	0.5 mL Triton X100

**Wash buffer III**	For 50 mL
10 mM Tris-HCl pH 8	0.5 mL Tris-HCl pH 8
1% IGEPAL CA630	0.5 mL IGEPAL (100%)
1% DOC (Sodium deoxycholat)	0.5 g
1 mM EDTA	0.1 mL 0.5 M EDTA pH 8
0.25 M LiCl	1.25 mL 10 M LiCl

**1 x TE**	For 50 mL
10 mM Tris-HCl pH 8	0.5 mL Tris-HCl pH 8
1 mM EDTA	0.1 mL 0.5 M EDTA pH 8

**Elution buffer**	For 25 mL
1% SDS	1.25 mL 20% SDS
0.1 M $NaHCO_3$	2.5 mL 1 M $NaHCO_3$

Table S2. Primers used for qPCR.

Locus	Forward primer	Reverse primer
Rpl32	TCA TTT CTC AGG CAC ATC TT	ACT CAC CGT AAA ACA GAT GG
Il4	TCT GCC TCC ATC ATC CTT CT	ACA CCA TAA TCG GCC TTT CA
Il5	ACC CTG AGT TTC AGG ACT CG	TCC CCA AGC AAT TTA TTC TCT C
Il10	CGA CCA GTT CTT TAG CGC TT	TGT GGC TTT GGT AGT GCA AG
Il17a	TGG TTC TGT GCT GAC CTC AT	GCT CTC CCT GGA CTC ATG TT
Gata3	CAC TCG GAT TCC TCT CTC CC	CCA GGA GAG GGG TCG TTT AA
Tbx21	AGA GAA AGC CCA GGA GCA G	TTC CAG CAG CCG TCG AAG
Rorc, i.2*	TGG GGT GCC TGT CAT CAT AC	TGA GAA CTT GGC TCC CTG TC
Foxp3	GAC TCA AGG GGG TCT CA	TTG GGC TTC ATC GGC AA
Ifng	CAT ACC CTT TCC TTG CTT TTC	TTG TGG GAT TCT CTG AAA GCA
Tnf	TGG GTT TCA GTT CTC AGG GT	GGG TTT GGA AAG TTG GGG AC
Tgfb	GCT CCC CTA TTT AAG AAC ACC C	CTC CCA AGG AAA GGT AGG TGA
Il4ra	TGG CTT GCA GAC TCA GAA GA	GGC TTG CCA TCC TGT TTT GA
Stat6	GTG TGG TTC AAT GGA GCT CC	TGT TTC CGG CTT CTC TGT CT
Cd40	TGG CTT TTG AGG TCC TGG AT	ATG GCT AGT CAC TGA GCA GG

All oligonucleotides were synthesized by Metabion (Planegg, Germany).

*RAR-related orphan receptor gamma, isoform 2 (RORgammaT) locus.

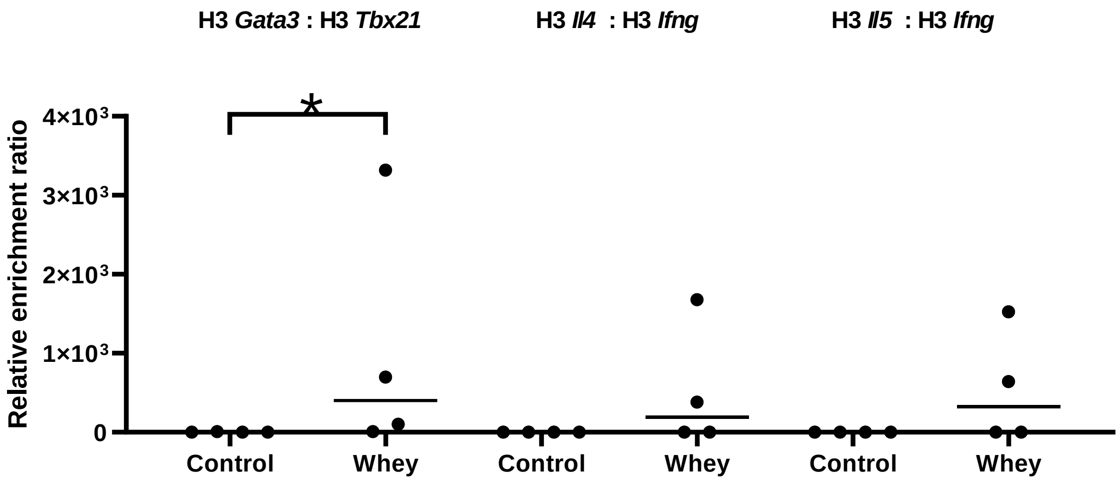

Figure S1. A confirmed allergic response to whey protein increased the ratio of histone H3 acetylation levels at the gene encoding Gata3 (*Gata3*) and the gene encoding T-box 21 (*Tbx21*) in spleen-derived $CD4^+$ T cells. Ratios of histone H3 acetylation levels at the *Gata3* locus and the *Tbx21* locus, the gene encoding interleukin 4 (*Il4*) and the gene encoding interferon γ (*Ifng*), and the gene encoding interleukin 5 (*Il5*) and *Ifng* in spleen-derived $CD4^+$ T cells of whey-sensitized mice and control mice upon challenge as determined by chromatin immunoprecipitation followed by qPCR. Data are presented as scatter dot plots showing individual data points and median. Comparisons were performed with a Mann-Whitney U test. *$p < 0.05$.
